# Current status of Complementary and Alternative Medicine Interventions in the Management of Pancreatic Cancer – An Overview

**DOI:** 10.1007/s11864-023-01146-4

**Published:** 2023-12-11

**Authors:** Aleksandra Tarasiuk, Grzegorz Mirocha, Jakub Fichna

**Affiliations:** Department of Biochemistry, Faculty of Medicine, Medical University of Lodz, Mazowiecka 5, 92-215 Lodz, Poland

**Keywords:** Pancreatic cancer, Treatment of pancreatic cancer, Complementary and alternative medicine, Traditional Chinese medicine

## Abstract

Pancreatic cancer (PC) remains the deadliest cancer worldwide. Most patients are diagnosed at the advanced or metastatic stage, leading to a poor prognosis. Awareness of the limitations of current therapy and accompanying pain, depression, malnutrition, and side effects of chemoradiotherapy may lead patients and physicians towards complementary and alternative medicine (CAM). CAM refers to a diverse set of medical and healthcare practices, products, and systems that are not part of conventional Western medicine. Despite the low-quality evidence supporting the efficacy of these methods, they remain appealing due to patients' beliefs, fear of death, and the slow development of conventional therapy. Hence, the possibility of using natural products for pancreatic cancer is increasing. CAM options such as: medical cannabis, plants, fungi, herbal formulas, and injections, which originate primarily from traditional Chinese or Japanese medicine i.e. Curcuma longa, Panax ginseng, Poria cocos, Hochuekkito, Juzentaihoto, and Rikkunshito, Shi-quan-da-bu-tang/TJ-48, Huang-qin-tang, Shuangbai San, Wen Jing Zhi Tong Fang, Xiang-Sha-Liu-jun-zi-tang, Aidi injection, Brucea javanica oil emulsion/Yadanziyouru injection, Compound Kushen injection, Huachansu injection, Kangai injection and Kanglaite injections are becoming promising candidates for the management of pancreatic cancer. The abovementioned substances/medications are the most popular or potentially effective in PC treatment and consequently CAM-based adjuvant therapy through improving patients’ quality of life, might be a useful addition in the treatment of pancreatic cancer patients.

## Introduction

Pancreatic cancer (PC) remains one of the most dangerous cancers worldwide. According to the Global Cancer Observatory (GLOBOCAN) 2020, the incidence was estimated at 495,773 patients in 2020 [1]. Different types of PC can be distinguished, such as pancreatic ductal adenocarcinoma (PDAC, 85% of cases) [2] or neuroendocrine tumors (less than 5% of cases) [3]. However, due to the enormous disproportion between the prevalence of these cancers, most data are related to PDAC. Around 80–90% of patients have unresectable tumors at diagnosis, and 52% of PC are already metastatic at presentation [4].

The limitations of currently used biomarkers and lack of typical clinical manifestations lead to minimal early detection of PC [5–7]. Carbohydrate antigen 19–9 (CA 19–9) is a gold standard biomarker for PC diagnosis with overall sensitivity in the range of 25% to 50% in early-stage disease and a sensitivity and specificity of 80% to 90% in patients with the symptomatic disease [8, 9]. However, CA 19–9 can be elevated in non-carcinogenic conditions such as benign biliary obstruction; moreover, it cannot be evaluated in 5–10% of the population due to a lack of genes encoding the Lewis blood group antigen.

PC remains the deadliest cancer worldwide, with an 11.5% of 5-year relative survival [10]. Based on current data, the PC is predicted to be the second leading cause of cancer death by 2030, despite its low incidence [11].

### Treatment options and challenges

Pancreatic cancer treatment may involve surgery, chemotherapy, radiation therapy, vaccination, pain management, immunotherapy, and dietary changes [12, 13].

Surgical management for PC depends on tumor location, with only 11% of patients having resectable or borderline resectable tumors [4]. Surgery offers curative potential, with a five-year relative survival of 44% [10]. Postoperative adjuvant chemotherapy (CT) is the current standard based on studies showing that gemcitabine plus capecitabine and FOLFIRINOX improve overall survival (OS) in PC patients [14, 15]. In contrast, neoadjuvant CT and radiotherapy (RT) before surgery are used in locally advanced pancreatic cancer (APC) and in most borderline resectable tumors to improve margin-negative resection rates and, as a result, increase the number of patients eligible for surgical treatment [16••, 17]. However, chemoradiotherapy can induce pancreatic fibrosis, potentially increasing complication rates [18].

Radiotherapy (RT) is a treatment for local APC, often combined with CT, immunotherapy, or surgery. It is recommended for patients with controlled disease after at least four months of CT [19]. Modern RT techniques like stereotactic body radiation therapy (SBRT) along with stereotactic MR-guided online adaptive radiation therapy (SMART) can improve the treatment of PC compared to conventional RT [20••]. However, RT's efficiency is limited due to pancreatic anatomy, organ motion, and contact with the GI tract; it also promotes cancer stem cell self-renewal [21].

Chemotherapy is the first-line treatment for unresectable and metastatic pancreatic cancer (MPC), with FOLFIRONOX or gemcitabine plus paclitaxel being the standard [16••]. PC cells are more resistant to gemcitabine, and patients with higher Physical Fitness Scores (PFS) should receive it for better curative effects. Tumor response to CT depends on factors like tumor microenvironment, genetic mutations, and tumor subtype [22]. Olaparib was approved as maintenance therapy in 2019 for patients with BRCA1 or BRCA2 mutations and MPC [23].

### Signs and symptoms

Pain is the most frequent symptom in PDAC, affecting almost 90% of patients. The origin of pain is various and contains visceral and neuropathic mechanisms such as pancreatic duct obstruction, tissue destruction, inflammation, and nerve infiltration [24]. Notable, severe abdominal pain with posterior radiation is associated with celiac invasion by the unresectable tumor, which is related to poor prognosis [25].

High levels of anxiety, despair, and decreased quality of life (QoL) are common in people with PC, but few studies have examined these outcomes for cancer patient-caregiver dyads [26–29]. According to one study [30], depression affects as many as 50% to 78% of people with PC, a percentage noticeably greater than that of patients with other GI malignancies. In another study [31], during the course of a 6-month treatment period, 22 patients with a confirmed diagnosis of PC had been screened positive for depression, anxiety, and sleep disruption. This may be associated with an awareness of the poor prognosis of the treatment and the short median overall survival (OS). However, like other symptoms, anxiety and depression significantly decrease QoL and performance status (PS) [32].

Because of the endocrine and exocrine functions of the pancreas, treatment of side effects related to PC impacts the nutritional status of patients. Malnutrition affects about 70% of patients, with the occurrence increasing with the PC stage [33]. Malnutrition is associated mainly with inflammation, which, through hypercatabolism, may lead to cachexia [34, 35]. Also, digestive tract stenosis, cholestasis, as well as diabetes mellitus are crucial challenges in nutrition management and might contribute to significant weight loss [33, 36, 37]. Malnutrition and cachexia negatively affect QoL, response to treatment, and survival [38, 39]. Interestingly, sarcopenia alone does not demonstrate a significant association with poor prognosis, although sarcopenic muscle proteolysis induces a substantial efflux of muscle amino acids, which may increase tumor progression [39–41].

Finally, PC is one of the most thrombogenic tumors – thromboembolic events during the disease occur in 20–35% of cases [24, 42]. Thromboembolic events which are serious complications of hospitalization are triggered by systemic inflammation and cancer cell secretion of factors that initiate the clotting cascade (e.g. microvesicle-tissue factor (MV-TF), factor VIII, von Willebrand factor) [43].

## Complementary and alternative medicine

According to the WHO, complementary and alternative medicine (CAM) refers to a broad set of healthcare practices originating from different cultures that are not part of that country’s own tradition or conventional medicine and are not fully integrated into the dominant healthcare system [44]. In this manuscript, we have focused on medical cannabis, plants, fungi, herbal formulas, and injections, which originate primarily from traditional Chinese or Japanese medicine. They are believed or have been evidenced to relieve pain and other symptoms, and perhaps effect cancer progression, as discussed below.

### Plant or fungi-derived compounds

*Curcuma longa* contains curcumin which is said to have antioxidant, anti-cancer, and anti-inflammatory effects. Curcumin may sensitize tumors to chemotherapeutic drugs used in PC, such as 5-FU, paclitaxel, gemcitabine, docetaxel, and oxaliplatin [45]. According to one study [46], it can improve radiosensitivity [47], as well as protect the healthy tissue from CT- and RT-induced toxicity. Curcumin, through various pathways, also demonstrates antimetastatic effects in GI cancers, including PC [48].However, those properties are evident only in *in vitro* models and based on current clinical trials, there is insufficient evidence to use curcumin as adjuvant therapy in PC [49]. Unfortunately, some severe and mild side effects of curcumin have been observed in the GI (e.g., GI hemorrhage, constipation, vomiting, abdominal pain) and cardiovascular systems. Thus, its application may be limited [50]. Poor performance in clinical trials may have resulted from curcumin’s low bioavailability [51]. Therefore, various curcumin analogues have been utilized with novel drug delivery systems (e.g., phospholipids, nanoparticles, and liposomes) [52]. Those preparations have shown significantly better performance and improved outcomes in various cancers [53].

For more than 2000 years, Chinese medicine principles have guided the use and practice of herb-herb or herb-drug combinations to improve therapeutic efficacy [54]. Herbs used in conjunction with anticancer medications have recently been shown to be capable of reversing chemoresistance caused by anticancer drug usage [55]. Thus, the utilization of herb-drug combinations to boost therapeutic impact, particularly in cancer treatment, is of significant interest. There are other plants that have been claimed to enhance cancer treatment, but none have garnered as much attention or a global reputation as ginseng. *Panax ginseng* (Ren-Shen, Korean Red ginseng) is a common TCM herb. Among various active components, ginsenosides such as Rb1, Rg1, and Rg3 seem responsible for *Panax ginseng* antitumor properties [52, 56]. According to a review on *in vivo* and *in vitro* studies by Xie J. et al. [57], conventional therapy with adjuvant treatment using ginseng and its ingredients has been reported to decrease the probability of PC recurrence. For example, neutral polysaccharide fraction (WGPN) prepared from *Panax ginseng* showed a synergistic effect with 5-FU improving its anticancer activity in Saroma-180, highly malignant mouse sarcoma cells, and reducing the harmful impact on the immune system caused by 5-FU in the mouse model [58]. In a randomized clinical trial from 2021 [59], patients who received Korean Red ginseng daily during adjuvant CT showed higher CD4 + T lymphocytes and CD4 + /CD8 + T lymphocyte ratio after CT. Moreover, in another clinical trial [60] ginseng has been also proposed to have beneficial effect in ameliorating cancer-related fatigue without significant toxicity Furthermore, it is believed to reduce the harmful effects of RT on normal tissues *in vitro* and *in vivo* due to its radio-protective properties [61].Of note, RN1 – an arabinogalactan polysaccharide isolated from the flower of *Panax notoginseng* – was showed to inhibit PDAC growth both *in vitro* and *in vivo* through the inhibition of galactin-3 (Gal-3) [62], which is associated with tumor progression [63]. It also promoted cell death through apoptosis and autophagy pathways, increasing chemosensitivity to gemcitabine *in vitro* [64].

*Poria cocos* is an essential medicinal and edible fungus that grows on pine trees. Its active components demonstrate anticancer, anti-inflammatory, and antioxidant potential *in vitro* [65]. A characterized mixture of triterpenes extracted from *P. cocos* and three purified triterpenes: pachymic acid (PA), dehydropachymic acid (DPA), and polyporenic acid C (PPAC), have been shown to suppress the proliferation of PC cells [66]. It has been emphasized that triterpenes from *P. cocos* inhibit the migration of PC cells associated with CDC20, which prevents metastases [67]. In other studies, pachymic acid (PA) has shown pro-apoptotic properties by targeting ER stress [68].

*Poria cocos* is increasingly used in Asian countries with the FOLFOX4 CT (therapy of colorectal cancer), enhancing treatment effectiveness and alleviating CT-related side effects according to a review and a meta-analysis of randomized-clinical trials [69, 70]. Hence, it is likely that it might have a similar impact on PC [71].

### Herbal formulas

*“Kampo”* medicine is a branch of CAM originating from Japan. For its three traditional preparations, *Hochuekkito*, *Juzentaihoto*, and *Rikkunshito*, many clinical trials have been published demonstrating beneficial effects for fatigue and cachexia treatment in various diseases and cancers, including PC [72]. Of interest, Fujitsuka N. et al*.*[73] presented beneficial effect of *Rikkunshito* on survival. Patients with PC who had ascites were given gemcitabine or gemcitabine plus *Rikkunshito*; in terms of stage and age, there was no significant difference between the two groups in baseline data. However, the administration of *Rikkunshito* dramatically increased the median survival of pancreatic cancer patients with ascites who were treated with gemcitabine. These findings suggest that *Rikkunshito*, through its dual action on ghrelin secretion and receptor sensitization, may be useful in clinical practice for cachectic cancer patients. A recent case series report has shown a superior median OS for *Kampo* medicine when used as an adjuvant to conventional therapy [74].

*Shi-quan-da-bu-tang* (SQDBT/TJ-48) is a formula of ten herbs. In the study by Ikemoto T. et al*.* its antitumor properties through increasing regulatory activities in T cells by decreasing Foxp3 + Treg populations in APC have been demonstrated [75]. It is also believed to reduce the side effects of CT and RT and surgical treatment as well as prevent from the development of metastasis [76]. According to a randomized, placebo controlled, preliminary study [77], SQDBT combined with *Juzentaihoto* (a single dose of 3 g, three times a day for four weeks) as adjuvant therapy increases QoL and decreases symptoms of anorexia [78, 79]. Additionally, SQDBT is said to alleviate CT-related anemia and fatigue [80].

*Huang-qin-tang* is a traditional Chinese herbal formula used to treat various GI ailments, such as diarrhea, nausea, vomiting, and abdominal cramps [46]. PHY906 is a modified pharmaceutical preparation derived from this formula, enhancing gemcitabine’s antitumor efficacy *in vivo* [81]. Clinical studies have indicated that PHY906 treatment might significantly decrease GI toxicity and alleviate CT-induced side effects, such as diarrhea, whereas PHY906 toxicity has not been observed [82].

*Shuangbai San* is a herbal preparation used topically to treat pain. A randomized, double-blind, placebo-controlled trial confirmed its effectiveness as a painkiller in mild pain in patients with liver cancer [83•]. Although there is no evidence for its use in PC treatment, it may constitute a new approach for cancer-related pain treatment in PC.

Long-acting oral morphine or other opioids are frequently used to treat PC pain. Patients who are unable to take opioids orally may be given continuous-release skin patches or suppositories. TCM may help patients recover from PC surgery and relieve symptoms such as pain as well as reduce the requirement for opioids and can be used alone or in combination with other anti-tumor drugs.

*Wen Jing Zhi Tong Fang* is a herbal formula known for its analgesic properties, which is used to alleviate cancer pain. Within the WHO three-step analgesic ladder, its application as a warm compress on the back is more effective than standard pain relief therapy [84]. This method is particularly useful in patients with intolerable side effects and addiction to analgesic drugs which has been emphasized in a comparative clinical trial by Cai P. et al*.* [84]. Unfortunately, the lack of data strictly referring to PC limits the applicability to PC. However, *Wen Jing Zhi Tong Fang* in combination with standard treatment could be considered as a potential pain intervention.

*Xiang-Sha-Liu-jun-zi-tang* (XSLJZT) is a well-known Chinese herbal formula. XSLJZT treats GI side effects by enhancing digestive tract motility and appetite, protecting against gastric mucosal injury, and improving the gastric accommodation reflex, which prevents dyspeptic symptoms [76]. On the basis of a nationwide population-based cohort study from 2018 [85], patients who had undergone XSLJZT adjuvant therapy for at least 90 days had significantly lower hazard ratios of mortality risk and higher survival probability.

### TCM injections

Aidi injection is an adjuvant TCM injection commonly used to treat various types of cancer, including PC. Propensity Score Matching Analysis by Xie G. et al*.* [86] emphasized that aidi injection combined with chemotherapeutic drugs can decrease the incident rate of damage to liver and kidney function, myelosuppression, and GI reactions caused by CT. Moreover, it has been proposed to improve QoL and PS [86]. Based on a network meta-analysis and network pharmacology, aidi injection can enhance clinical efficacy and safety of CT in treating PC [87••].

*Brucea javanica* oil emulsion injection (BJOEI) (or Yadanziyouru injection) is an extract of ripe fruit from *Brucea javanica.* Numerous studies have reported its anticancer properties *in vitro,* such as inhibition of angiogenesis, cell proliferation and invasion, induction of apoptosis, and autophagy [88]. In a study by Yang H. et al*.* [89], BJOEI enhanced gemcitabine efficacy in the patient-derived orthotopic xenograft (PDOX) mouse PC model, leading to increased survival. Of note, network meta-analysis by Zhang D. et al*.* [90] revealed that BJOEI combined with CT reduces adverse effects and improves QoL. Most clinical evidence of BJOEI efficacy in enhancing chemotherapy is limited to lung, liver, colorectal, gastric, and esophageal cancer [91•]. Nevertheless, a recent meta-analysis from 2022 by Wang H. et al*.* [92] provided the evidence which supports the fact that BJOEI combined with conventional chemotherapy may exhibit a statistically significant and clinically important effect in the improvement of overall response rate, clinical benefit rate, performance status, as well as reduced incidence of the following adverse drug reactions: neutropenia, leukopenia, nausea and vomiting, diarrhea, liver damage, hand-foot syndrome, and peripheral sensory nerve toxicity in patients with gastric cancer. Moreover, BJOEI has been shown to display the strongest analgesic effect in relieving cancer-related pain among TCM injections [93••].

Compound Kushen injection (CKi), a Chinese patent drug, is widely used as adjuvant therapy in the cancer treatment, especially neoplasms of the digestive system. Matrine and oxymatrine are believed to be the compounds of CKI that have anti-cancer properties [94]; the former inhibits proliferation, arrests the cell cycle, and induces apoptosis in PC *in vitro* [95]. In a meta-analysis from 2022 [87••] it has been emphasized that Cki combined with CT may improve OS and QoL as well as relieving CT-related thrombocytopenia, CT-related GI adverse effects, and pain when compared to CT alone [87••].

Huachansu injection (HCSi) is a water-soluble preparation extracted from the toad’s skin and parotid venom glands (*Bufo bufo gargarizans Cantor*) containing Chansu [46]. HCSi inhibits PC metastasis in mouse models of human tumor xenografts [96]. Moreover, the already cited meta-analysis by Wang H. et al*.* [87••] revealed that HCSi as adjuvant therapy in PC treatment improves clinical efficacy and reduces the adverse effects of CT.

Another report from the above-mentioned meta-analysis [87••] indicates that Kangai injection (Kai), a Chinese medicine widely used in cancer treatment, when used as adjuvant therapy in PC treatment, improves clinical efficacy by reducing tumor volume and improving PS.

Kanglaite injection (KLTi) is an acetone extract of herbal medicine coix seed (Semen *Coicis Yokuinin*) prepared as a herbal medicine using modern advanced pharmaceutical technology. In one study, its antitumor properties were thought most likely to result from intervening in the cell cycle, inducing apoptosis, and suppressing tumor growth [97••]. Patients who underwent KLTi (30g/day) combined with gemcitabine treatment had a significantly better progression-free survival than those who undergo standard therapy in an efficacy exploratory study. [98]. A PRISMA-compliant meta-analysis from 2019 [99] revealed that KLTi combined with CRT is more effective than CRT alone; additionally, it improves QoL and alleviates CRT-related adverse effects.

### Medical cannabis

Cannabis is a flowering plant genus including the species *sativa*, *indica, and ruderalis.* Cannabis contains three groups of bioactive molecules: flavonoids, terpenoids, and cannabinoids. The term “cannabinoids” relates to three groups of substances based on their source: phytocannabinoids (occurring in plants), endocannabinoids (occurring naturally in the human body), and synthetic cannabinoids. Delta-9-tetrahydrocannabinol (THC) and cannabidiol (CBD) are well-known, naturally occurring compounds of cannabis. Their beneficial properties are widely used in treating cancer pain, nausea and vomiting, mood and sleep disorders, and appetite stimulation [95, 100].

Cannabinoids show various anticancer effects on PC *in vitro* but there is no evidence for this in clinical studies to date. [101]. However, developing an effective form of administration seems to be a big challenge due to the cannabinoids’ low aqueous solubility and poor stability [102••]. Moreover, it has been observed that a significant first-pass effect in the body reduces their bioavailability in oral administration [103]. Hence, achieving therapeutic concentrations in a tumor’s environment is a challenge for conventional routes for drug delivery systems [104]. Therefore, we focus on using cannabinoids in palliative treatment in which observing the therapeutic/beneficial effect of cannabinoid use is feasible, “conventional” administration is possible, and self-treatment with cannabinoids may be expected in some patients. Importantly, although patients using cannabis report better influence of the plant extracts than from synthetic products, most research has been performed with the use of synthetic products due to the illegality of natural cannabis in most of the world [105]. Hence, we had to limit our discussion to cannabinoid-based medicines (CBMs) such as THC, cannabidiol, HU-211, ajulemic acid, for which legislation is becoming more prevalent worldwide [106, 107].

CBMs may be effective for the management of neuropathic and cancer-related pain treatment on the basis of some research, e.g. a double-blind, randomized, placebo-controlled, paralleled group study [107], and a review article from 2020 [108]. However, CBMs application is limited due to a lack of high-quality evidence of efficacy and potential harm [107]. According to an original study by Noyes R. et al*.* [109], high-dose THC treatment (20 mg dose/once a day) can reduce cancer pain, but it is as sociated with severe side effects. Although a 10 mg dose/once a day of THC is better tolerated, its analgesic effect is not strong enough to manage cancer pain [109].

CBMs may be helpful in chemotherapy-induced nausea and vomiting (CINV) related to CB1 receptor anti-emetic activity after binding THC [110, 111]. Although much evidence has proven clinical effectiveness of CBMs in CINV treatment [111, 112], there is a high risk of bias, which may decrease the quality of the evidence [113]. Of note, in a clinical trial by Lane M*. *et al*.* [114] dronabinol (THC) and prochlorperazine synergize in reducing CINV. Thus, CBMs combined with conventional therapy may constitute an evidence-based treatment rather than alternative therapy and should be considered by physicians if traditional methods fail [100, 115]. On the other hand, in a systemic review by Smith et al*.* [110], it has been emphasized that cannabis-based drugs may be beneficial in the treatment of chemotherapy-induced nausea and vomiting. However, the studies' methodological shortcomings restrict the results, and future research including current chemotherapy regimens and newer anti-emetic medicines is likely to alter these findings [110].

Although a randomized clinical trial from 2018 confirms that cannabinoids are effective against cancer-associated anorexia (that promotes the development of the anorexia-cachexia syndrome, which is related to poor clinical outcomes in lung cancer) [116], they appear to be less effective than conventional treatment [117, 118]. However, in a randomized, double-blind, placebo controlled pilot trial from 2011 [119] it has been revealed that administration of cannabinoids is associated with improved taste, smell, and food enjoyment.

CBMs may improve sleep quality; thus, they may constitute treatment for insomnia. Although data about their application in cancer-related insomnia is limited [117], in accordance with a systemic review and meta-analysis by Whiting P. F. et al. [113] CBMs administration in patients with chronic pain and multiple sclerosis has confirmed their positive influence.

Side effects such as disorientation, somnolence, euphoria, anxiety, hallucination, and nausea are primarily associated with THC [115, 120]. It has been even proposed that CBD is antagonistic, alleviating those side effects [121]. Notably, most CBMs contain CBD; thus, their application is safer than street cannabis which contains a high THC:CBD ratio [118]. Moreover, the prescription of cannabis should be limited in patients with heart diseases or those using cardiotoxic drugs due to a higher risk of myocardial infarction [122]. Smoked cannabis may induce respiratory side effects; thus, it should be limited in patients with lung diseases [123].

Patients may use cannabis by themselves, but the dose when self-applied is difficult to control and depends on various factors (e.g., breath hold or duration of inhalation). Furthermore, street marijuana, as mentioned before, contains high levels of THC, thus, its self-application can aggravate side effects. PC patients may be particularly prone to side effects due to the narrow therapeutic window associated with low weight, resulting from cachexia and malnutrition. Overall, patients are advised to use cannabinoids under close cooperation between patient and physician and—according to current evidence—each therapy should be arranged individually.

## Conclusions

Dr. Nina Sanford from UT Southwestern Medical Center, specializing in radiation oncology, together with her team, conducted a survey [124••] on usage of CAM in cancer on 3118 cancer patients. The results of the study showed that one third of the respondents use unconventional methods of treatment and many of them do not inform their doctor about it. This is of concern as supplements may affect CT and interfere in potentially harmful ways. Therefore, there are several factors to consider before taking herbal supplements. First, supplements may negatively affect CT and reduce the effectiveness of radiation therapy as radiation requires oxygen to work by creating free radicals. Taking supplements containing high amounts of antioxidants is believed to potentially reduce the effectiveness of radiation. Moreover, the effectiveness of many herbal supplements is not supported by solid, long-term research. The most alarming issue is that Chinese supplements are often not officially approved by any of the drug agencies (i.e., EMA – European Medicines Agency or FDA – US Drug and Food Agency). Finally, patients who believe a particular supplement can cure or help prevent cancer may avoid conventional cancer treatments altogether or delay recommended follow-up to monitor for recurrence [124••].

However, many patients still use CAM. The reasons for this phenomenon are many, including fear of serious illness and debilitating therapy, fear of death, faith in the power of nature, but also limited resources of conventional medicine and gaps in an inefficient health care system that does not always meet the expectations and needs of all patients with the disease. It is not uncommon for the family and relatives of the oncological patient to be the initiators of paramedical activities and alternative cancer therapies. However, supplements may give people false hope when they feel powerless as they offer apparent relief during a constantly stressful time for their mind and body. Furthermore, supplements often provide patients with promise a semblance of control over a condition that is not easily controlled.

On the other hand, according to a population-based cohort study from 2018 [85] adjunctive Chinese herbal medicine may help patients with PC live longer lives or at least improve their QoL. The most used single herb and Chinese herbal formula for the treatment of PC patients appears to be *Xiang-sha-liu-jun-zi-tang*. Other complementary and alternative medicine compounds, which could be considered as the potential treatment options or adjuvant therapy in PC patients have been summarized in Table 1. However, pharmacological studies or clinical trials to validate these findings are needed in the future.

**Table 1:** Summary of complementary and alternative medicine interventions and their proposed benefits on pancreatic cancer

	CAM COMPOUND OR PREPARATION	PROPOSED BENEFITS ON PC	REFERENCE
Plant- or fungiderived compounds	*Curcuma longa*	Sensitizes tumors to chemotherapeutic drugs Antimetastatic effect	[48]
			[51]
	*Panax ginseng*	Decreases the probability of PC recurrence, enhance treatment effectiveness	[58, 59]
		Ameliorates cancer-related fatigue	[60]
		Radio-protective effect	[61]
	*Poria cocos*	Inhibits the migration of PC cells	[67]
		Pro-apoptotic properties	[68]
Herbal formulas	*Kampo*	Decreases fatigue and cachexia	[73]
	*Shi-quan-da-bu-tang*	Increases regulatory activities in T cells Reduces side effects of CRT	[75] [80]
	*Huang-qin-tang* (PHY906)	Enhances gemcitabine's antitumor efficacy Decrease GI toxicity and alleviate CTinduced side effects	[81] [82]
	*Shuangbai San*	Pain relief	[83•]
	*Wen Jing Zhi Tong Fang*	Pain relief	[84]
	*Xiang-Sha-Liu-jun-zi-tang*	Prevents dyspeptic symptoms	[76]
TCM injections	*Brucea javanica* oil emulsion injection	Inhibits angiogenesis, cell proliferation and invasion, induces apoptosis and autophagy	[88]
	Compound Kushen injection	Inhibits proliferation, arrests the cell cycle, induces apoptosis	[95]
	Huachansu injection	Inhibits PC metastasis Reduces adverse effects as adjuvant therapy	[96] [93••]
	Kangai injection	Reduces tumor volume and improves PS Induces apoptosis, suppresses tumor growth	[98] [97••]
Medical cannabis	THC	Pain relief	[100]

CAM – complementary and alternative medicine; CRT – chemoradiotherapy, GI – gastrointestinal; RCT – randomized clinical trial; PC – pancreatic cancer, PS – performance status, TCM – Traditional Chinese Medicine, THC – Delta-9-tetrahydrocannabinol

In conclusion, there is often a nihilistic approach to PC, determined by poor prognosis and decades of failure of many clinical trials that showed promise in the early stages of research. However, recent years have brought several new therapeutic options. Although these are not “spectacular successes”, we are undoubtedly observing "step by step" an improvement in the prognosis in some groups of patients. Therefore, it is particularly important to try to individualize the therapy of this cancer when planning the treatment of patients with PC, with the aim of seeking the best solutions for PC patients.

## Abbreviations

5-FU: 5-Fluorouracil
APC: Advanced pancreatic cancer
BJOEI: *Brucea javanica* Oil emulsion injection
CAM: Complementary and alternative medicine
CBD: Cannabidiol
CBMs: Cannabinoid-based medicines
CINV: Chemotherapy-induced nausea and vomiting
CKi: Compound *Kushen* injection
CRT: Chemoradiotherapy
CT: Chemotherapy
DPA: Dehydropachymic acid
GI: Gastrointestinal
HCSi: *Huachansu* Injection
Kai: *Kangai* Injection
KLTi: *Kanglaite* Injection
MPC: Metastatic pancreatic cancer
OS: Overall survival
PA: Pachymic acid
PC: Pancreatic cancer
PDAC: Pancreatic ductal adenocarcinoma
PPAC: Polyporenic acid C
PS: Performance status
QoL: Quality of life
RT: Radiotherapy
SQDBT: *Shi-quan-da-bu-tang*
TCM: Traditional Chinese Medicine
THC: Delta-9-tetrahydrocannabinol
XSLJZT: *Xiang-Sha-Liu-jun-zi-tang*
